# Biological Characteristics and Clinical Outcome of Triple Negative Primary Breast Cancer in Older Women – Comparison with Their Younger Counterparts

**DOI:** 10.1371/journal.pone.0100573

**Published:** 2014-07-07

**Authors:** Binafsha M. Syed, Andrew R. Green, Christopher C. Nolan, David A. L. Morgan, Ian O. Ellis, Kwok-Leung Cheung

**Affiliations:** 1 School of Medicine, University of Nottingham, Nottingham, United Kingdom; 2 Department of Oncology, Nottingham University Hospitals, Nottingham, United Kingdom; H. Lee Moffitt Cancer Center & Research Institute, United States of America

## Abstract

Triple negative (ER, PgR and HER2 negative) breast cancers (TNBCs) are often considered as a poor prognostic phenotype. There is dearth of evidence showing the prevalence and biological behaviour of TNBCs in older women. This study aimed to analyse their biological characteristics in comparison with a well characterised younger series from a single centre with long term clinical follow-up. Over 37 years (1973–2010), 1,758 older (≥70 years) women with early operable (<5 cm) primary breast cancer were managed in a dedicated clinic and have complete clinical information available. Of these 813 patients underwent primary surgery and 575 had good quality tumour samples available for tissue microarray analysis using indirect immunohistochemistry. A total of 127 patients (22.1%) had TNBCs and full biological analysis of 15 biomarkers was performed. The results were compared with those of their younger (<70 years) counterparts 342 (18.9%) from a previously characterised, consecutive series of primary breast cancer treated in the same unit (1986–1998). The 127 older patients with TNBCs showed lower rates of Ki67 and CK 7/8 positivity and high rates of bcl2 and CK18 positivity when compared with their younger counterparts (p<0.05). There was no significant difference in the long term clinical outcome between the two age groups, despite the fact that 47% of the younger patients had adjuvant chemotherapy, while none in the older cohort received such treatment. EGFR, axillary stage and pathological size showed prognostic significance in older women with TNBCs on univariate analysis. Despite not having received adjuvant chemotherapy, the older series had clinical outcome similar to the younger patients almost half of whom had chemotherapy. This appears to be related to other biomarkers (in addition to ER/PgR/HER2) eg Ki67, bcl2 and cytokeratins which have different expression patterns influencing prognosis.

## Introduction

The risk of breast cancer increases with advancing age. [1]. Oestrogen receptor (ER) positive tumours become more common in older women [2], [3], suggesting a possible change in biology with age. Many studies have reported an inverse correlation between ER positivity and Human Epidermal Growth Factor Receptor-2 (HER2) expression [4], [5], [6], [7]. Thus a small proportion of older women would be expected to have triple negative (ER, progesterone (PgR) and HER2 negative) breast cancers (TNBCs).

It is generally believed that TNBCs tend to have a poor prognosis. Given the lack of conventional therapeutic targets i.e. hormone receptors and HER2, patients with TNBCs are left with chemotherapy as the only systemic therapy option, if this is required as an adjuvant treatment. Conversely, owing to limited physiological reserve and increased number of co-morbidities, some older women may not be able to tolerate chemotherapy and also the Oxford Overview has not yet had sufficient number of older women from randomised trials to demonstrate a definite and significant absolute benefit of chemotherapy in this age group [8]. Thus patients and clinicians both show reluctance to use chemotherapy, and its use in older women still remains debatable. A lot of research has recently been directed towards TNBCs but most of the studies are focused on younger patients with minimal or no representation of older women [9], [10], while those focusing on older women showed inconsistency in defining the age cut-off for including patients in the older cohort [11] This study aimed to analyse the biology and clinical outcome of TNBCs in older (≥70 years) women and compare the results with their younger counterparts.

## Methods

Over a period of 37 years (1973–2010) 1,758 older (≥70 years) women with early operable primary breast cancer were managed and followed up in a dedicated clinic, with availability of a complete set of clinical information from the date of diagnosis till death or last follow-up. Early operable primary breast cancer was defined clinically as tumour size of ≤5 cm, with no or palpable mobile ipsilateral axillary lymphadenopathy, without evidence of distant metastases (cT1-2, N0-1, M0). The patients were initially identified from the Histopathology archive of paper records (before 1987, N = 240) and a prospectively established computerised database (since 1987, N = 1,518). The clinical course of the disease was subsequently confirmed from the clinical notes. The dedicated clinic, which started off as a surgical clinic, had evolved into a combined surgical/oncology facility in the recent decade, following the same clinical guidelines at any time point. Although there has been a change in the management protocols over the period as previously described [12], most of the change was attributed to the use of endocrine therapy as far as systemic therapy was concerned. Therefore it would be unlikely that the changes had impacted on patients with TNBCs.

Among all these patients, 813 of them had surgery as their primary treatment. Of these, 575 had good quality tumour samples from their surgical specimens available for tissue microarray (TMA) construction. For the purpose of this study, the tumour samples were analysed using indirect immunohistochemistry (IHC) on TMAs for ER, PgR, HER2, p53, Cytokeratins (CK) 5/6, 7/8, 14, 17, 18, 19, Bcl2, E-Cadherin and Muc1. Assessment of Ki67 was done on whole tumour sections. Histological grade of the tumours was based on the original histology report, according to a uniform protocol using the Elston-Ellis modification of Scarff-Bloom Richardson (SBR) grading system after 1988 and SBR before 1988 [13].

### Younger series

In order to compare with the younger (<70 years) patients, patients with stage matched (cT1-2,N0-1,M0) TNBCs were retrieved from a previously characterised institutional database (Nottingham/Tenovus series) along with the data of the above mentioned biomarkers [14], [15], [16]. The Nottingham Tenovus series consists of clinical and biological data of younger (<70 years) patients with early operable primary breast cancer established in 1980s and tumours were prospectively analysed using IHC on TMAs, constructed using surgical specimens (N = 1809). As it was planned at the outset of the project to compare the present older series with the younger patients, the variables collected for the older series were the same as those collected for the younger patients and the methods for tumour analyses were the same.

### Tissue microarray construction

The TMAs were constructed following the previously described method [14], [17], where the most representative part of the tumour was identified and a core of 0.6 mm thickness was obtained and implanted into the TMA block, by using a manual tissue microarrayer (MP06 Beecher Instruments Inc, USA).

### Immunohistochemistry

Indirect IHC was performed using Streptavidin-Biotin Complex (ABC) method as previously described [14]. Briefly, TMA slides were incubated at 60° C for 10 minutes, followed by washing with two Xylene and three alcohol baths for 5 minutes and 10 seconds each respectively. The slides were treated with citrate buffer for 20 minutes to retrieve the antigen. Endogenous peroxidase activity was inhibited by 0.3% hydrogen peroxide. The slides were incubated for a specified time for the primary antibody at room temperature (details of the antibody dilution and incubation time are given in Table 1). The secondary antibody was applied for 45 minutes, followed by the treatment with diaminobenzidine and copper sulphate.

**Table 1:** A summary of the anti-bodies source, methods and cut-offs to define positivity of the markers used to analyse biology of early operable primary breast cancer in older women.

Biomarker	Antibody reference	Dilution	Incubation time	Antigen retrieval method	Cut –off to define positivity
ER	RM-9101-SP1/NeoMarkers	1∶100	45 minutes	Citrate Buffer	0
PR	PgR 636/Dako	1∶200	45 minutes	Citrate Buffer	0
HER2	Hercep test Kit- Dako	Pre-diluted	30 minutes	Not required	3+
Ki67	M 7240/Dako	1∶100	45 minutes	Citrate Buffer	10
P53	DO-7/Novocastra	1∶100	45 minutes	Citrate Buffer	5
CK5	M26/Thermo Scientific	1∶100	45 minutes	Citrate Buffer	10
CK5/6	M7237/Dako	1∶100	45 minutes	Citrate Buffer	10
CK7/8	34779/BioSciences	1∶100	45 minutes	Citrate Buffer	10
CK14	LL002/Vector Laboratories	1∶100	45 minutes	Citrate Buffer	10
CK17	E3/Vector Laboratories	1∶100	45 minutes	Citrate Buffer	10
CK18	M7010/Dako	1∶100	45 minutes	Citrate Buffer	10
CK19	CM 242A/Biocare Medical	1∶100	45 minutes	Citrate Buffer	10
Bcl2	M0887/Dako	1∶100	45 minutes	Citrate Buffer	10
E-Cadherin	M3612/Dako	1∶100	45 minutes	Citrate Buffer	30
Muc1	NCL/Novocastra	1∶300	45 minutes	Citrate Buffer	20
EGFR	31G7/Zymed	1∶30	60 minutes	Proteinase K	0

### Immunohistochemical scoring

The IHC staining of the biomarkers was assessed by the percentage of cells stained as well as semi-quantitatively by McCarty's histochemical scoring (H-score) [18] method. For intra-observer reproducibility the cores were scored thrice (by BMS) and the average of the score was taken as the final score and for inter-observer concordance an expert pathologist blinded from the scores and the clinical data, scored 10% of cores. There was high inter and intra-observer concordance between the scorers (Kappa score ≥0.7).

Table 1 summarises the definitions of positivity of different biomarkers using pre-defined cut-offs.

### Statistical methods

Comparison was made between the two age groups <70 and ≥70, using Chi-square test and time dependant variables were compared using Kaplan-Meier method with application of Log-rank and generalised Wilcoxon tests as appropriate for statistical significance. A p-value<0.05 was considered significant. Given the difference in length of follow-up of the two series, analysis was carried out according to time periods and comparison was done at five years from the date of diagnosis to avoid any potential bias. Breast cancer specific survival measures biological behaviour of the disease without being influenced by deaths due to causes other than breast cancer. As the older women are at higher chance of presenting with a number of co morbidities and a considerable proportion is life threatening [19], and lead to the higher rate of non breast cancer deaths [12], [20], thus overall survival does not accurately measure virulence of tumours as it takes all causes of death into account. Therefore the two groups were compared in terms of breast cancer specific survival (defined as deaths due to breast cancer), local recurrence (defined as the re-appearance of the tumour in the same breast after wide local excision or in the mastectomy flap), regional recurrence (defined as appearance of the tumour in ipsilateral axilla) and metastases (defined as distant recurrence including bone, lung, liver, and also supraclavicular lymph nodes). Multivariate analysis was carried out using Cox-regression model. The Predictives Analytic Software (PASW 18, Chicago, Illinois) was used for data collection and analysis.

### Ethical approval

The work presented in this manuscript was part of a study entitled ‘Development of a molecular genetic classification of breast cancer' approved by the Nottingham Research Ethics Committee (approval number C1080301), who waived the need for written informed consent from the participants.

## Results

Among all age groups 469 patients had TNBCs including 342 in women <70 years (342 out of 1809, 18.9%) and 127 in older (≥70 years) women (127 out of 575, 22.1%). Basic demographic characteristics of the two age groups are summarised in Table 2. Older women with TNBCs were significantly of larger pathological size and lower grade, but there were no significant differences in histological type and axillary stage.

**Table 2:** A summary of the general characteristics and biology and management pattern of triple negative breast cancer – young (<70 years) versus older (≥70 years).

Character	All N(%)	<70 years	≥70 years	p-value
		N(%)	N(%)	
**Median age (range)**	57.5 (25.91)	51(25–69)	75(70–91)	
**Histological types**				
**Ductal carcinoma (no special type)**	383(83.3)	276(82.4)	107(85.6)	0.12
**Tubular carcinoma**	12(2.6)	8(2.4)	4(3.2)	
**Lobular carcinoma**	8(1.7)	3(0.9)	5(4.0)	
**Other types**	57(12.4)	48(14.3)	9(7.2)	
**Pathological size**				0.002
**≤2 cm**	208 (45.4)	169(49.6)	39(33.3)	
**>2 cm**	250(54.6)	172(50.4)	78(66.7)	
**Axillary lymph node status**				0.61
**No positive**	263(62.2)	213(62.5)	50(61.0)	
**1–3 positive**	111(26.2)	91(26.7)	20(24.4)	
**≥4 positive**	49(11.6)	37(10.9)	12(14.6)	
**Grade**				0.007
**1**	8(1.8)	5(1.5)	3(2.8)	
**2**	45(10.0)	26(7.6)	19(17.4)	
**3**	396(88.2)	309(90.9)	87(79.8)	
**Ki67 positive**	303(75.4)	243(87.7)	60(48.0)	<0.001
**p53 positive**	242(52.7)	188(55.6)	54(44.6)	0.02
**E-Cadherin positive**	374(80.6)	284(84.0)	90(71.4)	0.002
**Bcl2 positive**	149(60.3)	57(43.5)	92(79.3)	<0.001
**Muc1 positive**	310(75.8)	215(75.2)	95(77.2)	0.37
**CK5/6 positive**	221(48.0)	160(46.8)	61(51.7)	0.20
**CK7/8 positive**	436(94.0)	328(95.9)	108(88.5)	0.005
**CK14 positive**	138(31.2)	97(28.7)	41(39.0)	0.03
**CK17 positive**	54(23.8)	25(22.5)	29(25.0)	0.38
**CK18 positive**	297(66.6)	195(60.2)	102(83.6)	<0.001
**CK19 positive**	401(86.6)	295(86.5)	106(86.9)	0.52
**Management pattern**				
**Surgery without adjuvant chemotherapy**	296(66.4)	169(53.0)	127(100)	
**Surgery with adjuvant chemotherapy**	150(33.6)	150(47.0)	0	

### Biological features

Comparison of the biomarkers between the two age groups showed that TNBCs in older women had significantly lower expression of Ki67, more normal p53 and higher expression of Bcl2 than younger women (Table 2). There was mixed luminal and basal cytokeratin expression with significantly higher expression of CK14 and CK18, but a reduction in the expression of CK7/8. There was no difference in the expression of CK17, CK5/6, CK 19 or Muc1.

### Management pattern and clinical outcome

A considerable proportion (47%) of younger (<70 years) patients received adjuvant chemotherapy as compared to their older counterparts where none of them received chemotherapy (Table 2).

**Figure 1:**
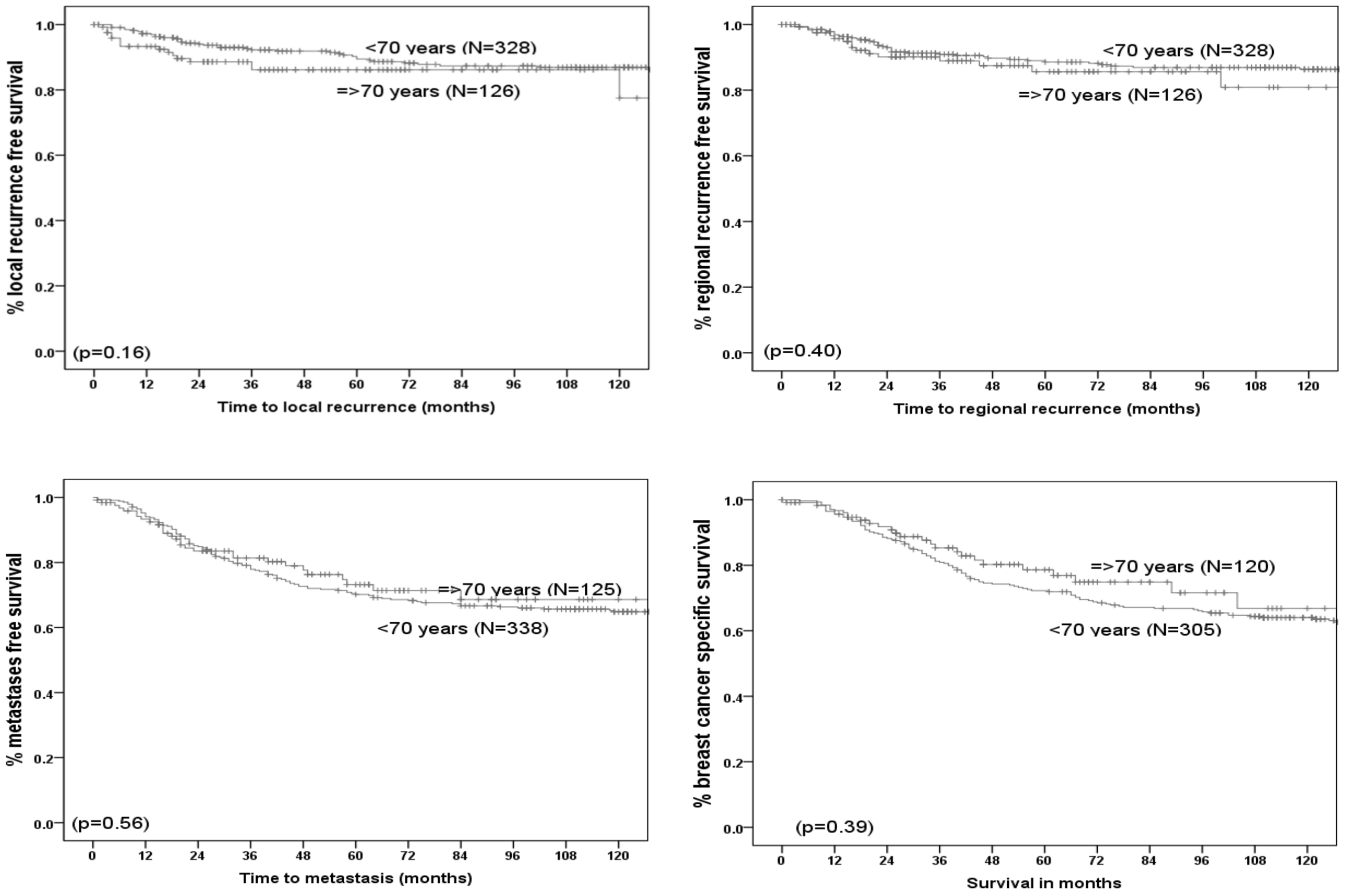
Pattern of clinical outcome of triple negative early operable primary breast cancer - Younger (<70 years) versus older (≥70 years).

At a median follow-up of 46 (longest = 204) months in the older women and 119 (longest = 135) months in the younger patients, there was no significant difference in the clinical outcome of the disease, in terms of recurrence and survival (Table 3, Figure 1). However there was a trend of even better survival in the older women (5-years breast cancer specific survival in <70 = 73% versus 79% in older patients), though it did not reach statistical significance. The 5-year rates of local and regional recurrences in younger patients were 10% and 9% versus 14% and 14% respectively in older patients. The rates of metastases were 30% and 27% in younger and older groups respectively.

**Table 3:** A summary of the clinical outcome of triple negative early operable primary breast cancer- younger (<70) versus older (≥70) patients.

Outcome measure	5-year rates in two age groups (%)		p-value
	<70 years	≥70 years	
**Breast cancer specific survival**	73	79	0.39
**Local recurrence free survival**	90	86	0.16
**Regional recurrence free survival**	91	86	0.40
**Metastases free survival**	70	73	0.56

The clinical outcome remained similar (ie. no difference) when comparison was made with stratification according to histological grade (p-value>0.05) (Data not shown).

### Prognostic factors

**Figure 2:**
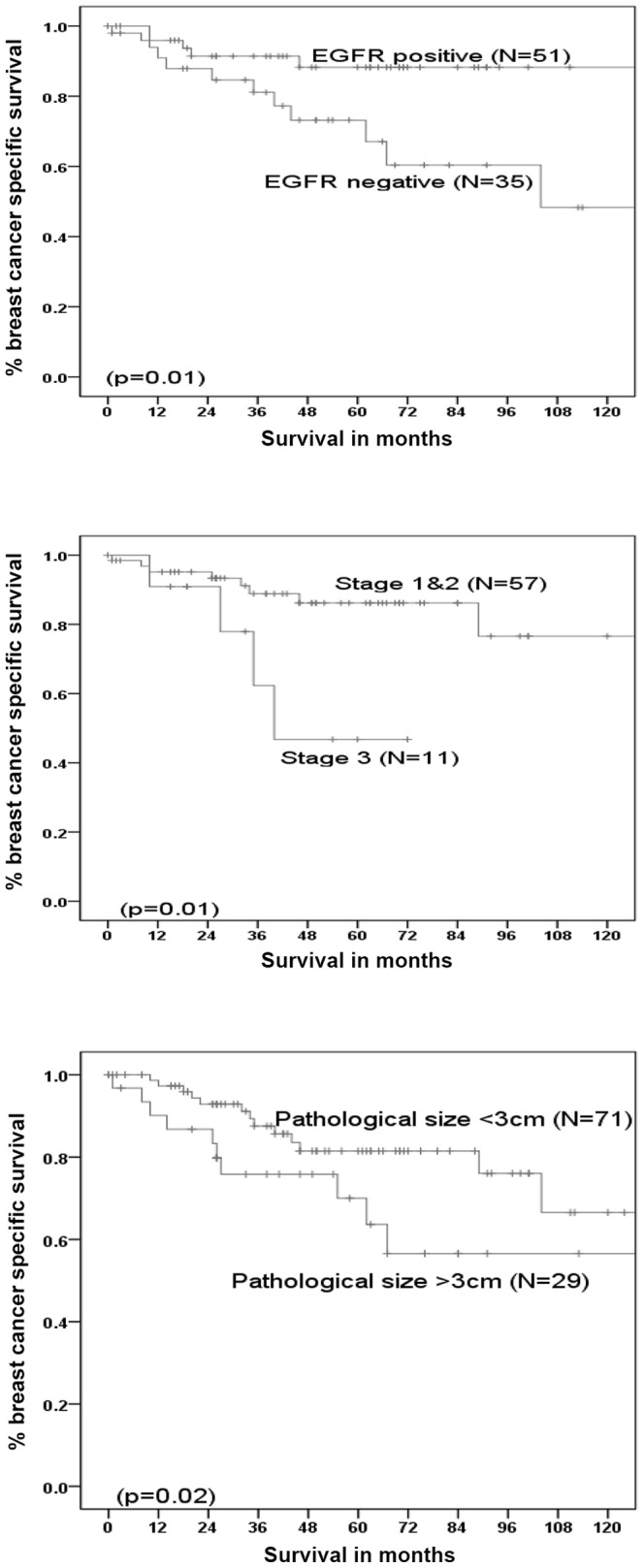
Prognostic factors showing significance influence on the breast cancer specific survival of older women with early operable triple negative breast cancer.

All the studied biomarkers were analysed for prognostic significance in the older series. Only EGFR showed prognostic significance in terms of breast cancer specific survival, along with stage and pathological size (Figure 2). Patients with EGFR positive tumours, axillary stage I or II disease, or pathological size <3 cm had significantly better breast cancer specific survival. However on multivariate analysis none of them showed any prognostic significance.

## Discussion

The results of the study show that TNBCs in older women appear to have less aggressive biology as compared to their younger counterparts by having more low grade tumours, reduction of Ki67 expression, more frequent normal p53 and higher expression of Bcl2. There appears to be no significant difference in the clinical outcome in the two age groups despite the fact that a higher proportion of the younger patients received chemotherapy. Positive EGFR status, lower axillary stage (<4 positive nodes positive) and small pathological size (<3 cm) were associated with significantly better outcome in older women with TNBCs, on univariate analysis.

The age cut-offs defining the older population remain inconsistent across various studies eg 55, 60, 65, 70 or 75 years have all been used. We defined older population in our study as women ≥70 years of age. This age cut-off has been previously reported to have an association with the beginning of a rapid decline of physiological reserve [21]. Also this is the same age cut-off used by our unit when the Nottingham Prognostic Index was devised and in a few other randomised controlled trials comparing surgery and primary endocrine therapy in older women [22], [23]. It would therefore appear to be reasonable to use such cut-off for the ease of comparability.

Overall TNBCs comprise 10–25% of the breast cancer population regardless of age [9], [24], [25], [26]. There is dearth of knowledge regarding the pattern of TNBCs with advancing age. In our study the tumour analysis was carried out using surgical specimens where there was a natural selection of more ER negative tumours for primary surgery as those older women with ER positive tumours who presented with a number of co morbidities and/or refused to have surgery had an option of primary endocrine therapy. An audit of a recent consecutive series of older women (part of this larger series) where the status of ER, PR and HER2 was determined on needle core biopsies as part of standard pathology report at the time of their diagnosis showed 13.4% TNBCs [12]. Thus the higher number of TNBCs in older patients in this study (22.1%) probably reflects the higher ER negative tumours within the series.

This study has presented comparison of biology and clinical outcome of this apparently more aggressive phenotype of breast cancer from a large series in two age groups. Overall there was a higher rate of grade 3 tumours, high proliferative index, mutant p53, which is in keeping with the literature [25], [26], [27]. In this study, TNBCs in the younger patients showed aggressive biology with higher rate of grade 3 tumours, Ki67 expression and p53 mutations as seen in many other studies which included younger patients. However none of these studies compared the two age groups. There was a mixed picture of luminal (CK7/8 and 18) and basal (CK 14 and 17) markers. In the older women studied here, there was a high expression of luminal CK 18, which has never been reported in the literature and this may have an impact on the possible better clinical outcome as seen here. However the rate of CK7/8 positivity was higher in younger patients. The study showed a high expression of basal markers, in keeping with the literature [25], [27] though it was even higher in older series which might be due to the higher representation of ER negative tumours in older women where there is a frequent association with basal cytokeratins [28], [29].

The overall outcome of the two age groups with TNBCs was poor with a 5-year breast cancer survival of 73% and 79% in <70 and ≥70 years age groups respectively, which is in keeping with the literature [30], as compared to that reported by us (5-year breast cancer survival being 91% and 90% respectively for the whole series of older women and in the ER positive subgroup) [12], [20]. However, despite the fact that they did not receive any adjuvant chemotherapy, the older women did not do any worse than their younger counterparts. As described above, this could be explained biologically due to a preponderance of tumours with lower grade, low proliferation index, normal p53, high Bcl2 proteins and high expression of luminal cytokeratins which all have been shown to be associated with better clinical outcome. There is therefore an urgent need of further studies to investigate this so that clinicians would know how best to select older patients for adjuvant chemotherapy. This is especially important in those older patients with poorer physiological reserve and significant co-morbidities. There could be a valid reason based on tumour biology for not recommending chemotherapy in some of these patients.

The expression of EGFR showed an association with better outcome. This has been reported in the literature where others have demonstrated better outcome of basal like tumours as compared to pure triple negative/quadruple negative (Quadruple negatives defined as the tumours negative for ER, PgR, HER2 and CK5/6 expression) tumours [9]. The risk of dying from breast cancer in the reported study was 43% higher in the group with negative CK5/6 and EGFR expression within triple negative group as compared to the patients who had CK5/6 and EGFR positive expression [9]. In addition, studies have shown that EGFR when co-expressed with luminal cytokeratins tends to be associated with a better prognosis [31]. This might be a possible explanation of the better outcome associated with EGFR positivity seen in the present study, where a high expression of CK18 was present in most cases. The presence of EGFR overexpression is also a potential good news as anti-EGFR immunotherapy (e.g. cetuximab and eroltinib) could be exploited. There is a strong need of inclusion of older women in clinical trials with anti-EGFR therapies to assess their clinical effectiveness as well as safety profile.

## Conclusions

Despite not having received adjuvant chemotherapy, the older series had clinical outcome similar to the younger patients almost half of which had chemotherapy. This may be due to other biomarkers (in addition to ER/PR/HER2) which have different patterns in these age groups influencing prognosis. The place of adjuvant chemotherapy in the treatment of these patients has yet to be identified.
